# CeRNA network analysis and functional enrichment of salt sensitivity of blood pressure by weighted-gene co-expression analysis

**DOI:** 10.7717/peerj.7534

**Published:** 2019-09-13

**Authors:** Han Cao, Han Qi, Zheng Liu, Wen-Juan Peng, Chun-Yue Guo, Yan-Yan Sun, Christine Pao, Yu-Tao Xiang, Ling Zhang

**Affiliations:** 1 Department of Epidemiology and Health Statistics, School of Public Health, Capital Medical University, and Beijing Municipal Key Laboratory of Clinical Epidemiology, Beijing, China; 2 The National Clinical Research Center for Mental Disorders & Beijing Key Laboratory of Mental Disorders & the Advanced Innovation Center for Human Brain Protection, Beijing Anding Hospital, School of Mental Health, Capital Medical University, Beijing, China; 3 Science Department, Peking University People’s Hospital, Beijing, China; 4 Department of Psychiatry, University of North Carolina at Chapel Hill, Chapel Hill, NC, United States of America; 5 Unit of Psychiatry, Institute of Translational Medicine, Faculty of Health Sciences, University of Macau, Macau, China

**Keywords:** Competitive endogenous RNAs, Gene set enrichment analysis, Long non-coding RNAs, Salt sensitivity of blood pressure, Weighted gene co-expression network analysis

## Abstract

**Background:**

Salt sensitivity of blood pressure (SSBP) is an independent risk factor for cardiovascular disease. The pathogenic mechanisms of SSBP are still uncertain. This study aimed to construct the co-regulatory network of SSBP and data mining strategy based on the competitive endogenous RNA (ceRNA) theory.

**Methods:**

LncRNA and mRNA microarray was performed to screen for candidate RNAs. Four criteria were used to select the potential differently expressed RNAs. The weighted correlation network analysis (WGCNA) package of R software and target miRNA and mRNA prediction online databases were used to construct the ceRNA co-regulatory network and discover the pathways related to SSBP. Gene ontology enrichment, gene set enrichment analysis (GSEA) and KEGG pathway analysis were performed to explore the functions of hub genes in networks.

**Results:**

There were 274 lncRNAs and 36 mRNAs that differently expressed between salt-sensitive and salt-resistant groups (*P* < 0.05). Using WGCNA analysis, two modules were identified (blue and turquoise). The blue module had a positive relationship with salt-sensitivity (*R* = 0.7, *P* < 0.01), high-density lipoprotein (HDL) (*R* = 0.53, *P* = 0.02), and total cholesterol (TC) (*R* = 0.55, *P* = 0.01). The turquoise module was positively related with triglyceride (TG) (*R* = 0.8, *P* < 0.01) and low-density lipoprotein (LDL) (*R* = 0.54, *P* = 0.01). Furthermore, 84 ceRNA loops were identified and one loop may be of great importance for involving in pathogenesis of SSBP. KEGG analysis showed that differently expressed mRNAs were mostly enriched in the SSBP-related pathways. However, the enrichment results of GSEA were mainly focused on basic physical metabolic processes.

**Conclusion:**

The microarray data mining process based on WGCNA co-expression analysis had identified 84 ceRNA loops that closely related with known SSBP pathogenesis. The results of our study provide implications for further understanding of the pathogenesis of SSBP and facilitate the precise diagnosis and therapeutics.

## Introduction

Salt sensitivity of blood pressure (SSBP), first proposed by Kawasaki in 1978 (Kawasaki et al., 1978), is defined as the blood pressure of some people exhibiting changes parallel to changes in salt intake (Elijovich et al., 2016). Hypertension that related with SSBP is called salt sensitive hypertension (SSH) (Mo & Ren, 2012). SSBP is one of the early damage markers of hypertension and an independent risk factor for cardiovascular morbidity and mortality (Franco & Oparil, 2006), especially for Asian populations (Kario et al., 2018). Understanding the key regulatory factors and pathogenic pathways of SSBP can help us screen the high-risk individuals and conduct personalized treatments.

Evidences have showed that microRNAs (miRNAs) (Moazed, 2009) are involving in the pathogenesis of SSH. For example, miR-429 (Zhu et al., 2017), miR-133a (Guo et al., 2014) and miR-29b (Liu et al., 2010) are differentially expressed in SSH rat model, and hsa-miR-361-5p is associated with SSH patients (Qi et al., 2017). However, there lacks evidences about the associations between long non-coding RNAs (lncRNAs) (Wang & Chang, 2011) and SSBP. A study revealed that the expression level of lncRNA sONE is significantly decreased in rat model of SSH (Zhang et al., 2015). It is necessary to explore the expression profile of lncRNAs in SSBP individuals. In addition, with the rising advent of the competitive endogenous RNA (ceRNA) theory, it has been discovered that lncRNAs and miRNAs function together to form biological modules (Xu et al., 2011). LncRNAs could competitively combine with the miRNA response element (MRE) and repress miRNA’s negative regulation of target mRNAs. This lncRNA-miRNA-mRNA triple network has been demonstrated in several diseases, but not in SSBP (Chen et al., 2018; Pan et al., 2017b; Xie et al., 2017; Yuan et al., 2017; Zhang et al., 2016). Thus, it is important to elucidate the ceRNA co-regulatory of SSBP with bioinformatic analysis and data mining, which could construct the systematic regulatory network and explore the interactive relationship of different biomarkers.

Weighted correlation network analysis (WGCNA) is a systematic biology-based approach that transforms gene expression profiles into co-expressed gene modules, which provides insight into biological pathways (Langfelder & Horvath, 2008). WGCNA has been widely applied in biological areas such as acute myeloid leukemia (Pan et al., 2017a), hepatocellular carcinoma (Pan et al., 2016), schizophrenia (Ren et al., 2015), as well as in coronary artery disease (Yan, 2018). Similarly, gene set enrichment analysis (GSEA) is a gene expression analysis method that creates *a priori* gene sets according to prior knowledge about biological states and can be used to gain biological insight into the transcript level (Subramanian et al., 2005).

Taken together, this study aims to screen for SSBP associated differentially expressed hub lncRNAs and mRNAs using WGCNA method, construct the co-expression network and ceRNA network, and provide insight into the mechanisms of SSBP on transcriptome level.

## Material and Methods

### Study population

Participants were selected from the System Epidemiology Study on Salt Sensitivity of Blood Pressure (EpiSS) study (Qi et al., 2018), which was registered in the WHO International Clinical Trials Registry Platform (No: ChiCTR-EOC-16009980). Salt-sensitive (SS) individuals were regarded as cases and salt-resistant (SR) ones were controls. The participants were invited by general practitioners of health community centers and underwent the modified Sullivan’s acute oral saline load and diuresis shrinkage test (MSAOSL-DST) and physical examination. The MSAOSL-DST is a diagnostic test used to distinguish SS from SR by orally administering 1,000 mL 0.9% saline solution and measuring the changes in blood pressure. MSAOSL-DST has been widely used in the definition of salt sensitivity with advantages of better acceptability and robust reproducibility. Individuals with more than 5 mmHg increase after salt-loaded and 10 mmHg decrease after oral administration of furosemide are diagnosed as SS, and others are SR (Li, Liu & Yang, 1994; Mu et al., 1993; Sullivan, 1991). To ensure the quality of subjects, only the subjects who had the same salt-sensitive diagnostic results as their records in the EpiSS database were included in this study. Blood was collected by professional nurses from the community health centers using PAXgene® blood RNA tube (PreAnalytiX, Hombrechtikon, Switzerland) and stored at −80 °C for further processing. This study was approved by the Ethical Committee of Capital Medical University, in compliance with the Declaration of Helsinki. All participants signed informed consent before the study began.

### RNA extraction and ceRNA expression profile

Total RNA was extracted and purified with PAXgene™ Blood RNA Kit (Cat#762174, QIAGEN, GmBH, Germany) following the manufacturer’s instructions. Qualified RNA was amplified and transcribed into cRNA, which was purified and hybridized to the SBC human ceRNA array V1.0 (Biotechnology Corporation, Shanghai, China) in Agilent G2545A Hybridization Oven. Slides were washed and then scanned by Agilent G2565CA Microarray Scanner with default settings. Raw data were normalized by the Quantile algorithm of the “limma” package of R 3.2.2 software (Ritchie et al., 2015).

### Screening differentially expressed lncRNAs (DE-lncRNAs) and mRNAs (DEGs)

Independent two-sample *t*-test was performed to analyze the DE-lncRNAs and DEGs between SS and SR after the normalizing of microarray data. Four inclusion criteria were applied to obtain the DE-lncRNAs and DEGs: a. fold-change >2 or <0.5; b. *P*-value <0.05 (two-tailed); c. expression levels were different compared to background signal values; d. RNAs with an expression difference between all samples that were higher than the median of all expression differences for each RNA, or, RNAs with a mean expression between all samples that were higher than the median of all expression differences for each RNA (Prieto et al., 2008). The R package “heatmaps” was used to draw the heatmaps for DE-lncRNAs and DEGs (Perry, 2019).

### WGCNA co-expression analysis

The lncRNA-mRNA co-expression network was analyzed by the “WGCNA” package of R 3.2.2 software (Langfelder & Horvath, 2008). Firstly, hierarchical clustering analysis was conducted to remove samples that were outliers based on Euclidean distance; then, we created a weighted adjacency matrix and calculated the soft thresholding power *β*. After this, a topological overlap matrix (TOM) was generated to describe the connections between genes. Gene modules were identified by cluster dendrogram analysis, with different colors representing different modules. We correlated clinical traits such as gender, age, salt sensitivity, and hypertension with each gene module to get the eigengene. Gene significance and module membership were calculated within modules to discover the most important transcripts.

### Target genes prediction

The prediction of lncRNAs to target miRNAs was conducted using the LNCipedia (http://lncipedia.org/db/search) and miRDB (http://www.mirdb.org/custom.html) databases. LNCipedia was utilized to obtain the full sequence of lncRNAs. Then, the sequences were imputed into miRDB and obtained the target miRNAs according to the principle complementary base pairing. The top five target scores were chosen as prediction results. The prediction of miRNAs to mRNAs was conducted with miRmap online database (http://mirmap.ezlab.org/) (Vejnar, Blum & Zdobnov, 2013). We chose the top ten as prediction results. The reason for selecting the top RNAs is to screen the most significant target prediction genes and remove the irrelevant RNAs according to the comprehensive ranks of “ΔG open”, probability exact, conservation “PhyloP” and miRmap score.

### CeRNA regulatory network visualization

We integrated the results of co-expression and prediction by using intersection elements of mRNAs. Then, the lncRNA-miRNA-mRNA triple competing relationship of each module was visualized and reconstructed with Cytoscape 3.4.0 (Langfelder & Horvath, 2008). In the network, nodes represented RNAs while lines represented co-expression and prediction relationship.

### mRNA functional enrichment analysis

Gene ontology (GO) and Kyoto Encyclopedia of Genes and Genomes (KEGG) pathway analysis of the mRNAs were performed using package “clusterProfiler” of R 3.4.3 software (Yu et al., 2012). Gene Symbol were transformed into Entrez Gene ID using bioDBnet online database (https://biodbnet-abcc.ncifcrf.gov/db/db2dbRes.php) for further GO and KEGG analysis.

### GSEA enrichment analysis

All the mRNA transcripts of the microarray were used to perform the GSEA enrichment analysis (Mootha et al., 2003). After ranking the expression level of mRNAs in SS and SR, we compared our ranking results with H and C2 gene sets of Molecular Signatures Database (MSigDB). We identified the enrichment function of these mRNAs according to their position in different pathways and calculated the false discovery rate (FDR). Gene sets with FDR <0.05 were regarded as significant. Similar gene sets. Enrichment results of data mining and GSEA were compared to GO and KEGG to determine the optimal functional analysis strategy.

## Results

### Differentially expressed lncRNAs and mRNAs

**Figure 1:**
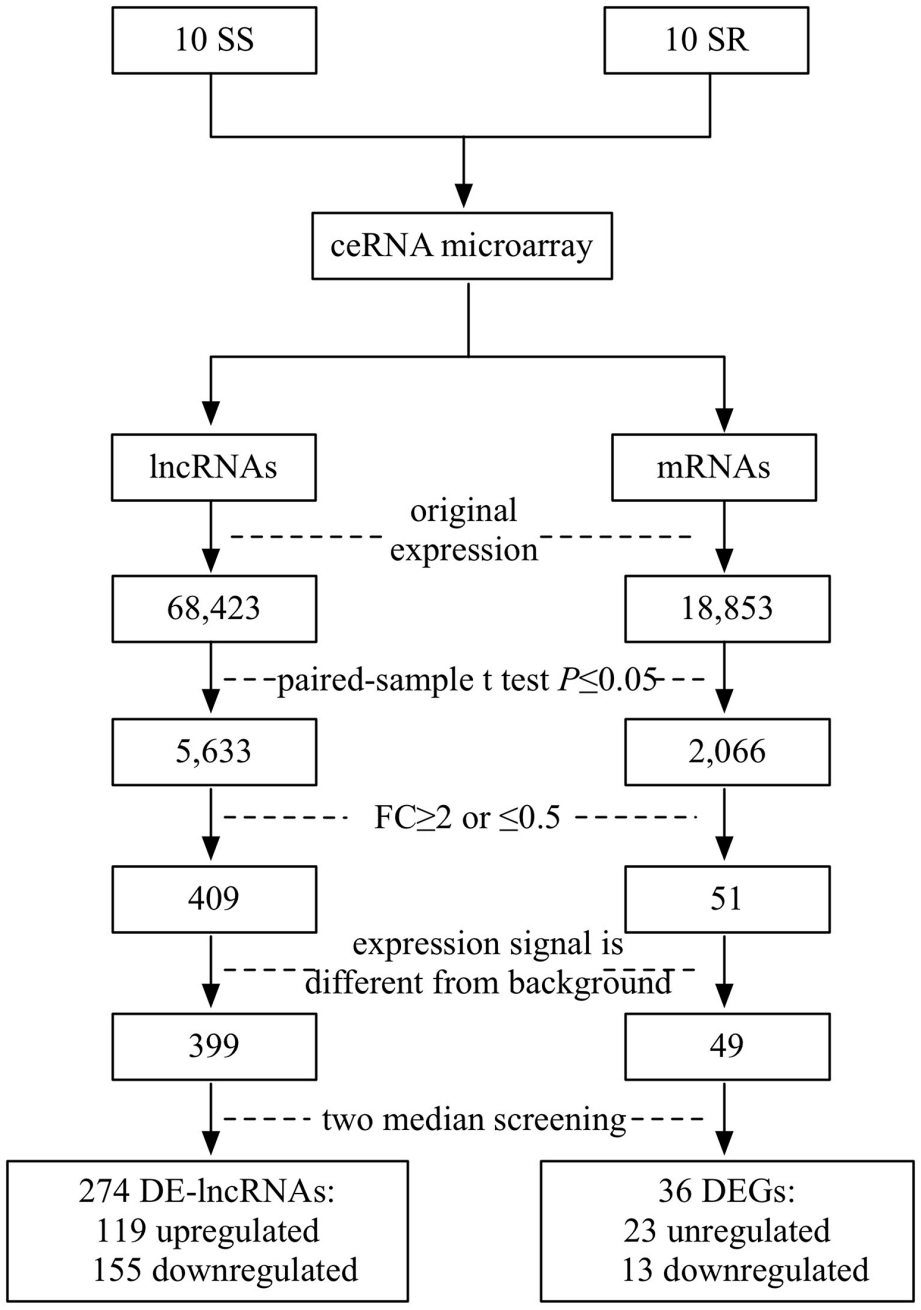
Flowchart of the screening of differentially expressed lncRNAs and mRNAs. SS, salt sensitive; SR, salt resistant; FC, fold change.

SBC ceRNA array v1.0 contained 68,423 lncRNAs and 18,853 mRNAs. Figure 1 describes the screening procedures of lncRNAs and mRNAs. According to the four inclusion criteria, we discovered 274 DE-lncRNAs (119 upregulated and 155 downregulated) and 36 DEGs (23 upregulated13 downregulated) (Supplementary Fig. 1).

### Baseline characteristics of patients

Ten SS and 10 SR were included in this study from two health community centers of Beijing between June 2016 and January 2017. Participants included eight males and 12 females, including 10 hypertensive patients and 10 normotensives. The average age of samples was 63.85 ± 0.47 years old. TG was noted to be significantly higher in SR compared to SS (*P* = 0.029). There was no significant difference in gender, age, TC, HDL, LDL, or GLU between groups (*P* > 0.05) (Supplementary Table 1).

### LncRNA-mRNA co-expression network construction

**Figure 2:**
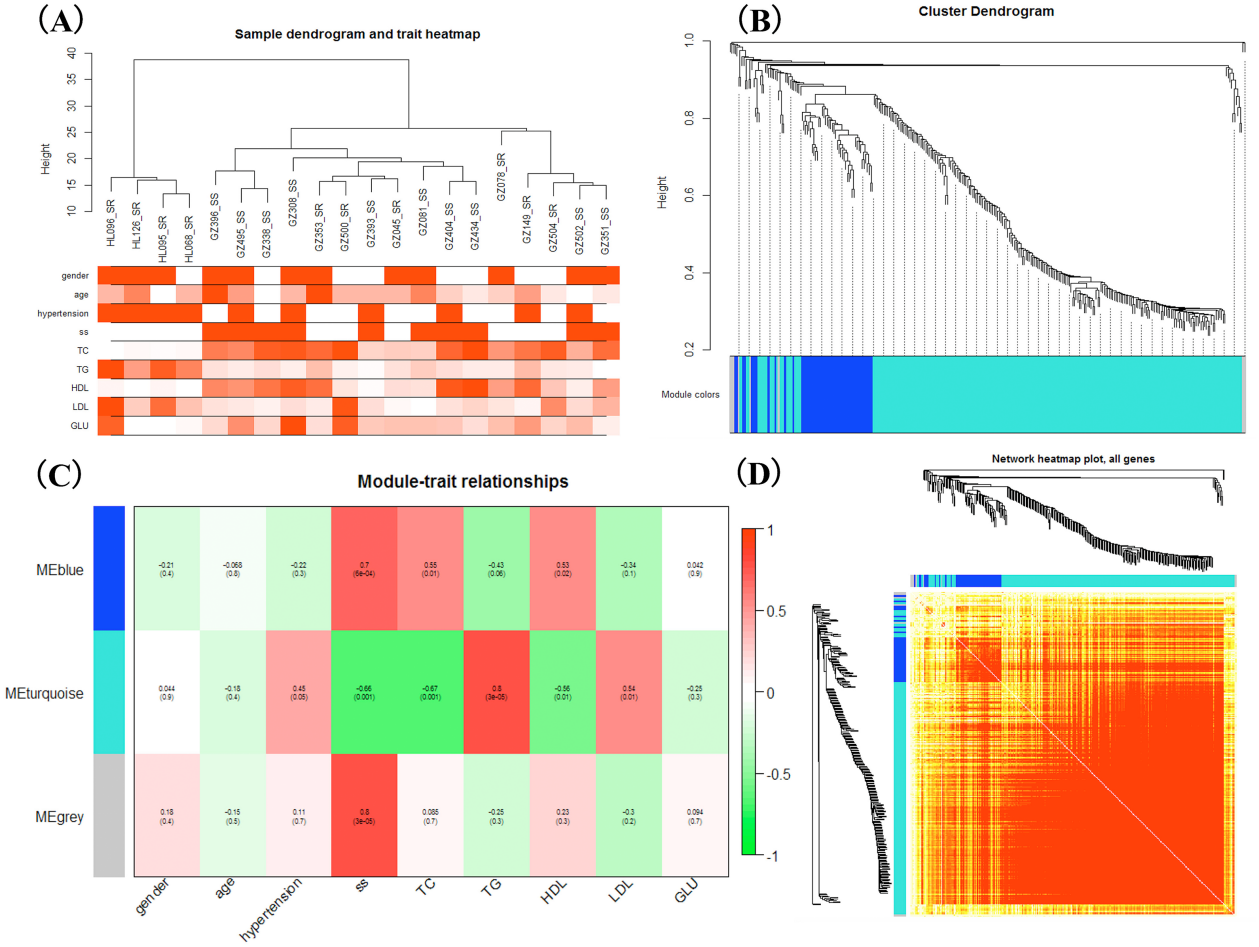
Weighted gene co-expression network analysis. (A) Sample dendrogram and clinical trait heatmap; (B) Hierarchical clustering. The branches of the tree represent the clusters of DE-lncRNAs and DEGs. Colors below the tree were gene modules that correspond to the clusters. (C) The correlation between gene modules and traits, and red represents a positive correlation and green represents a negative correlation; (D) LncRNA-mRNA co-expression network modules. Light color represents low overlap and darker red means higher overlap between RNAs. lncRNAs and mRNAs were organized into two modules.

**Figure 3:**
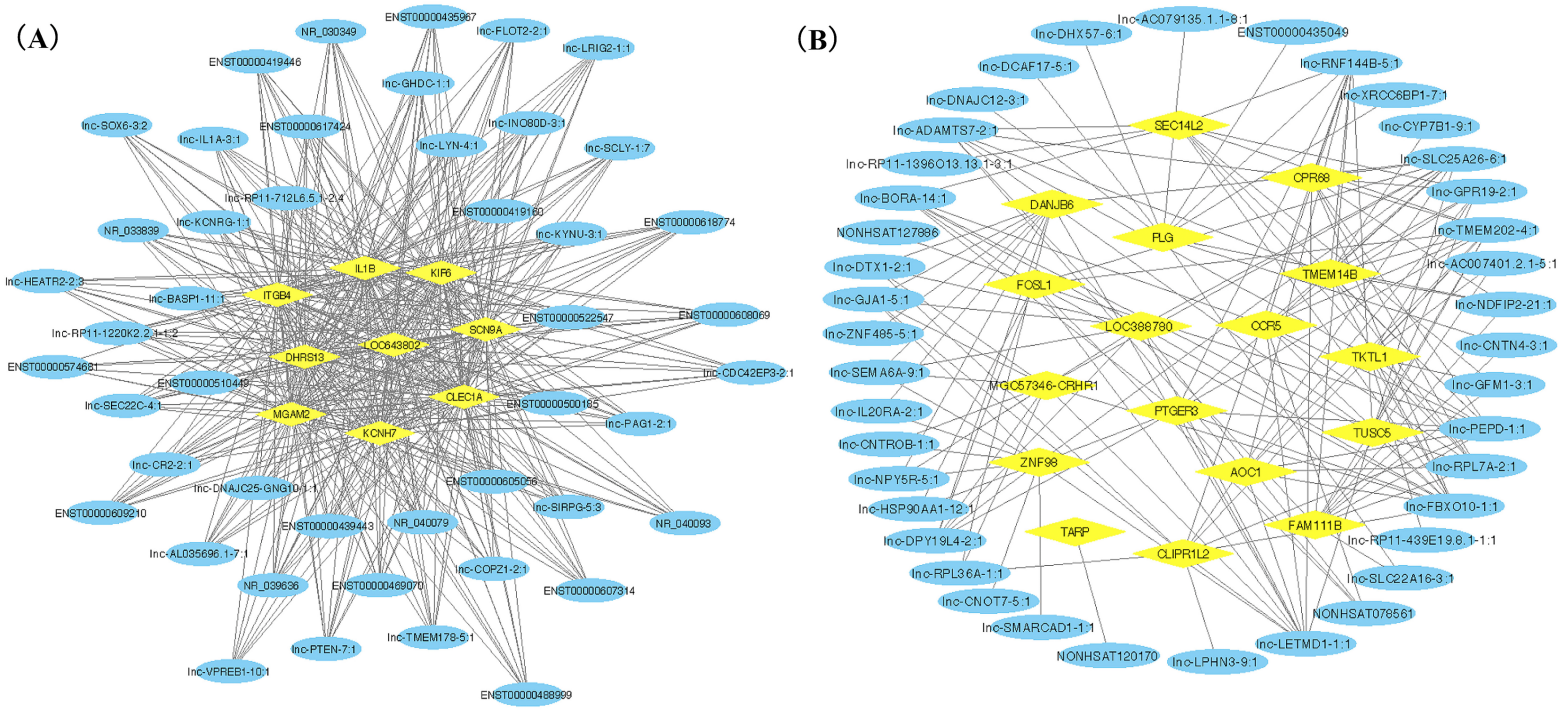
LncRNA-mRNA co-expression network plots of two gene modules. Yellow ovals represent hub mRNAs and blue triangles represent lncRNAs. (A) Co-expression network of lncRNAs and mRNAs in blue module; (B) Co-expression network of top ten lncRNA and mRNAs in turquoise module.

WGCNA was applied to detect the potential interactions between lncRNAs and mRNAs. Sample dendrogram analysis showed no outliers, so all 20 samples were included for further analysis. We could know from Fig. 2A that SS subjects were related with higher TC, HDL and lower TG. We used *β* = 6 to get adjacency matrix and generated a TOM dendrogram after calculating scale independence and mean connectivity (Fig. 2B). The cluster dendrogram shows three colors of modules (blue, turquoise and grey). The blue module contained 46 lncRNAs and 9 mRNAs and the turquoise module contained 191 lncRNAs and 17 mRNAs. Grey module was unclassified genes. Thus only blue and turquoise modules were considered for further analyses. The top 10 weighted lncRNAs and mRNAs in blue and turquoise co-expression network modules were summarized in the Supplemental Information (Tables S2 and S3). The correlations between modules and clinical traits are depicted in Fig. 2C. We found that the blue module had a positive relationship with salt-sensitivity (*R* = 0.7, *P* < 0.01), HDL (*R* = 0.53, *P* = 0.02), and TC (*R* = 0.55, *P* = 0.01). The turquoise module was positively related with TG (*R* = 0.8, *P* < 0.01) and LDL (*R* = 0.54, *P* = 0.01). A cluster heatmap was performed to comprehensively reflect the adjacency and topological structures of genes (Fig. 2D). We found two clusters in the heatmap, which represent the two modules. Then, the co-expression network was visualized with Cytoscape software (Figs. 3A and 3B). Due to too many nodes, only top ten associated lncRNAs of each mRNA were performed in turquoise co-expression network (Fig. 3B).

### CeRNA network construction

**Figure 4:**
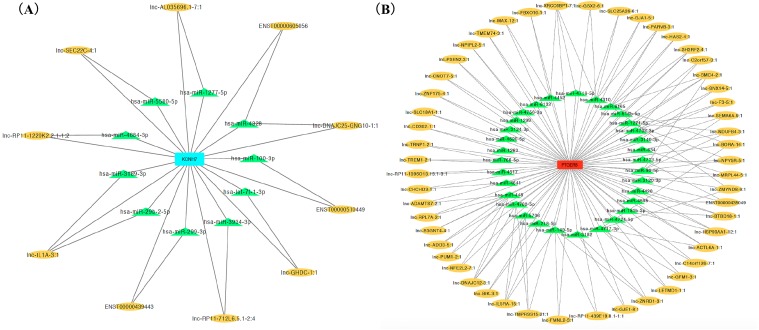
CeRNA network of salt sensitivity of blood pressure of blue modules (A) and turquoise modules (B). Red and blue diamonds in the center of network represent *PEGER3* from turquoise module and *KCNH7* from blue module, respectively; green triangles signify miRNAs and yellow ovals denote lncRNAs.

We ranked lncRNAs by module membership in blue and turquoise modules. LncRNAs with *P* < 10^−7^ were selected for target miRNAs and mRNA prediction using miRDB and miRmap databases. Finally, 17 of 46 (37.0%) lncRNAs from the blue module and 128 of 191 (67.0%) lncRNAs from the turquoise modules were chosen for further prediction analysis because their full sequences could be obtained from LNCipedia. After target genes prediction, 79 miRNAs and 220 mRNAs were included in the blue module, while 347 miRNAs and 607 mRNAs were included in the turquoise module. Then, we merged the mRNAs from prediction with co-expression and got 9 (*MGAM2, ITGB4, KCNH7, CLEC1A, KIF6, LOC643802, DHRS13, IL1B, SCN9A*) overlapping mRNAs in the blue module and 14 mRNAs (*TARP, GPR68, CCR5, TUSC5, ZNF98, TMEM14B, FOSL1, PLG, FAM111B, DNAJB6, SEC14L2, PTGER3, GLIPR1L2, TKTL1*) overlapping in the turquoise module. Due to *PTGER3* and *KCNH7* were associated with known SSBP mechanism pathways, such as sodium reabsorption and potassium ion-channel, which cause fluctuations of blood pressure (Kelly & He, 2012), these two DEGs were selected to perform ceRNA network of two modules (Figs. 4A and 4B). There were 10 miRNAs and 10 mRNAs in blue turquoise module, which forming 11 ceRNA loops. Similarly, there were 32 miRNAs and 51 lncRNAs in turquoise module, which forming 73 ceRNA loops.

### Gene ontology and KEGG pathway analysis

All of the DEGs coming from co-expression and prediction were united for genes annotation enrichment analysis to investigate the potential functions of these differentially expressed lncRNAs and mRNAs. For the turquoise module, the most significant cellular component was the synaptic membrane. Axon guidance, neuron projection guidance, regulation of short-term neuronal synaptic plasticity and response to nicotine were the top four significant for biological processes (*P* < 0.001); RNA polymerase II core promoter proximal region sequence-specific DNA binding was the most significant for molecular functions (*P* < 0.001). For the blue module, the most significant cellular components were the synaptic vesicle membrane and exocytic vesicle membrane (*P* < 0.001), and the most significant molecular function was transcriptional activator activity (*P* = 0.007). The results of KEGG pathway analyses showed that the most significant pathway for the turquoise module was aldosterone synthesis and secretion; whereas for the blue module, the most significant pathway was that of PI3K-Akt signaling (Table 1).

**Table 1:** Top 10 significant KEGG pathways for blue and turquoise modules.

Modules	KEGG ID	Description	*P* -value	*Q* -value	Gene count
Blue	hsa04151	PI3K-Akt signaling pathway	0.001	0.107	11
	hsa05165	Human papillomavirus infection	0.001	0.107	10
	hsa04926	Relaxin signaling pathway	0.002	0.107	6
	hsa04713	Circadian entrainment	0.003	0.107	5
	hsa04115	p53 signaling pathway	0.005	0.107	4
	hsa05020	Prion diseases	0.005	0.107	3
	hsa05214	Glioma	0.006	0.107	4
	hsa05212	Pancreatic cancer	0.007	0.107	4
	hsa05218	Melanoma	0.007	0.107	4
	hsa04512	ECM-receptor interaction	0.009	0.107	4
Turquoise	hsa04925	Aldosterone synthesis and secretion	0.000	0.019	11
	hsa04918	Thyroid hormone synthesis	0.001	0.073	8
	hsa04962	Vasopressin-regulated water reabsorption	0.001	0.073	6
	hsa04916	Melanogenesis	0.002	0.073	9
	hsa04261	Adrenergic signaling in cardiomyocytes	0.003	0.073	11
	hsa04911	Insulin secretion	0.003	0.073	8
	hsa05031	Amphetamine addiction	0.003	0.073	7
	hsa04727	GABAergic synapse	0.004	0.073	8
	hsa04728	Dopaminergic synapse	0.004	0.073	10
	hsa04360	Axon guidance	0.004	0.073	12

### GSEA enrichment of all mRNAs in ceRNA array

When H hallmark gene sets were regarded as reference sets, 37 of 50 genes sets were upregulated in SS, and three of them were significantly different (*P* < 0.05) (Fig. S2). Table 2 summarizes the significant SS-related leading edge genes sets. Thirteen gene sets were upregulated in the SR group; two gene sets, pancreas beta cells and epithelial mesenchymal transition, were significantly expressed (*P* < 0.05). For KEGG database of C2 gene sets (curated gene sets), 124 pathways were upregulated in SS and ten of them were significantly different (*P* < 0.05). The different pathways involved processes like glycan biosynthesis, propanoate metabolism, melanoma, aminoacyl trna biosynthesis, chronic myeloid leukemia, renal cell carcinoma, and cytokine receptor interaction. Fifty-three pathways were upregulated in SR; out of those, seven were significantly expressed, including pathways for olfactory transduction, ribosome, steroid hormone biosynthesis, primary bile acid biosynthesis, drug metabolism cytochrome P450, calcium signaling pathway, and metabolism of xenobiotics by cytochrome P450.

**Table 2:** Salt sensitivity related significant leading edge genes sets (hallmark).

Gene sets names	Counts	ES	NES	*P-* value	FDR *q*-value
UNFOLDED_PROTEIN_RESPONSE	112	0.401	1.489	0.006*	0.312
HEDGEHOG_SIGNALING	35	0.469	1.446	0.035*	0.236
PEROXISOME	103	0.358	1.318	0.040*	0.575
PROTEIN_SECRETION	95	0.349	1.263	0.103	0.709
ALLOGRAFT_REJECTION	200	0.308	1.232	0.081	0.735
REACTIVE_OXIGEN_SPECIES_PATHWAY	47	0.369	1.184	0.220	0.914
IL6_JAK_STAT3_SIGNALING	87	0.319	1.153	0.207	0.982
MITOTIC_SPINDLE	198	0.286	1.151	0.160	0.866
IL2_STAT5_SIGNALING	198	0.282	1.121	0.199	0.947
DNA_REPAIR	142	0.293	1.121	0.212	0.853
UV_RESPONSE_DN	143	0.286	1.106	0.254	0.860
NOTCH_SIGNALING	32	0.361	1.080	0.343	0.924

**Notes.**

ES enrichment score NES normalized enrichment score FDR false discovery rate

## Discussion

In this study, 274 DE-ncRNAs and 36 DEGs were identified through differential analysis. WGCNA analysis discovered the correlation between clinical traits and three modules (turquoise, blue and grey), and turquoise and blue modules were selected for enrichment analysis. Eighty-four ceRNA loops were identified and lnc-CD302-1:1 →hsa-miR-1283 →*PTGER3* may be of great importance for participating in mechanism of SSBP.

GO and KEGG analyses revealed that the turquoise module was related with aldosterone synthesis and secretion, which was one of the pathogenesis pathways of SSBP (Dengel et al., 2001). Aldosterone is a kind of steroid hormone and mainly responsible for fluid homeostasis of the body. Excess aldosterone would result in the reabsorption of salt and water and thereby the elevation of blood pressure (Shibata & Fujita, 2011). The role of aldosterone is more obvious in SSH patients, who have already undergone damages of kidney and exhibit overproduction of aldosterone. The lncRNAs and mRNAs that we screened out may involve in renin-angiotensin-aldosterone (RAAS) pathway through regulating the expressions of corresponding genes.

Among the 14 mRNAs of turquoise ceRNA network, prostaglandin E receptor 3 (*PTGER3*) plays a prominent role in the cardiovascular system. They involved in biological activities such as the regulation of blood pressure, metabolism of sodium and water, inflammation, reangiostenosis, and so on (Ceddia et al., 2016; Grilo et al., 2011). Among the 32 miRNAs that had predictive relationships with *PTGER3*, hsa-miR-1283 was associated with essential hypertension. Yang et al. (2015) found that hsa-miR-1283 may regulate the expression of *ATF1* through binding with the 3′UTR of the gene, which was abnormally expressed in essential hypertension. Due to SSBP is a clinical trait of hypertension, hsa-miR-1283 is probably associated with SSBP. Although there is no direct evidence showing that lnc-CD302-1:1 is directly associated with SSBP, the co-expression of lnc-CD302-1:1 and *PTGER3* as well as the predictive relationship of lnc-CD302-1:1 to hsa-miR-1283 provide hypothesis for future investigation.

In blue module, PI3K-Akt signaling pathway was the most significant pathway for SSBP. Insulin could activate the PI3K-Akt pathway by stimulating nitric oxide (NO) synthesis and thereby promoting vasodilatation (Kobayashi et al., 2004). Insulin sensitivity is also related with salt sensitivity by hyperinsulinemia, over-activation of sympathetic nervous system, and reducing suppression of RAAS pathway (Yatabe et al., 2010). Thus, the active of PI3K-Akt pathway may play a protective role in prevention and treatment of SSBP, corresponding to the strong correlation of the blue module and SSBP.

GSEA showed that SSBP is mainly associated with basic biological processes including glycan biosynthesis, and inositol phosphate metabolism. However, the results of GO and KEGG that based on WGCNA screening showed more SSBP-relevant pathways. So, the process of screening the differentially expressed RNAs, WGCNA co-expression analysis, and target genes prediction could effectively discover the accurate pathway information for SSBP, and more importantly, explore the function of ceRNA theory in the pathogenesis of SSBP. As all the mRNAs are considered when conducting GSEA analysis, the results of GSEA could be more comprehensive and therefore helpful for discovering the unknown pathways. However, there are some limitations for the two methods. For WGCNA, the nodes with low connectivity degree would not be recognized by modules. The heterogeneities of samples would result in the difficulties in module recognition and sample size under 15 could not use WGCNA (Liu et al., 2017). For GSEA, although it is a group-set analysis method, there are a few false positives due to focusing on sample-level variations rather than pathway-levels (Yi & Stephens, 2008). Thus, new methods are necessary to be developed to overcome the limitations. For this study, the combination of WGCNA data mining with GSEA could screen for key regulators of diseases and provide ideas for exploring pathogenesis pathways of other diseases.

There are a few limitations in our study. The regulatory effects of lncRNA to miRNA and miRNA to mRNA need to be further validated by real-time polymerase chain reaction with a larger sample size and functional experiments. The representative of participants is limited because they are older age and living in Beijing. In addition, whether the microarray data mining process is applicable for other diseases is currently unknown and should be explored in future studies.

## Conclusions

The microarray data mining process based on WGCNA co-expression analysis had identified 84 ceRNA loops for SSBP. The results of our study provide implications for understanding the pathogenesis of SSBP and facilitate the precise diagnosis and therapeutics.

##  Supplemental Information

10.7717/peerj.7534/supp-1 Supplemental Information 1 Miame Checklist

10.7717/peerj.7534/supp-2 Supplemental Information 2 Supplementary Materials

10.7717/peerj.7534/supp-3 Supplemental Information 3 WGCNA code

## Additional Information and Declarations

### Microarray Data Deposition

The following information was supplied regarding the deposition of microarray data: The microarray data is available at GEO: GSE135111.

### Data Availability

The following information was supplied regarding data availability: The raw data is available at GEO: GSE135111.
