# Supplementary material for: CeRNA network analysis and functional enrichment of salt sensitivity of blood pressure by weighted-gene co-expression analysis
Source: PeerJ. 2019 Sep 13;7:e7534. doi: 10.7717/peerj.7534 (PMC6746216; doi:10.7717/peerj.7534)
Supplement: Supplemental Information 1 [file peerj-07-7534-s001.docx]

**Miame Checklist**

**Part 1 Experiment description**

In the ceRNA microarray, 10 salt-sensitive individuals were regarded as cases and 1o salt-resistant individuals were controls. The whole blood samples were collected by professional nurses in the morning after fasting for 8 hours. There were no interventions on participants before collecting the blood samples.

**Part 2 Array design**

The SBC human ceRNA array V1.0 (Biotechnology Corporation, Shanghai) was performed to detect the expression levels of lncRNAs and mRNAs. There are 68,423 lncRNAs and 18,853 mRNAs on this array. The length of probe is 60nt, and the probes are designed according to human/GRCh38(hg38).

**Part 3 Samples**

Total RNA was extracted from whole blood samples. Then, NanoDrop ND-2000 spectrophotometer and Agilent Bioanalyzer 2100 were applied to inspect RNA concentrations and integrations. Qualified RNA was amplified and transcribed into cRNA. Labeled cRNA was purified by RNeasy mini kit (Cat.# 74106, QIAGEN, GmBH, Germany).

**Part 4 Hybridizations**

In the hybridization, firstly we need to prepare the 10X blocking agent, then, we mixed the Cyanine 3-labeled cRNA, 10X blocking agent, nuclease-free water and 25X fragmentation buffer and placed the samples on ice after 13,000 centrifuge. The samples need to be manually added in clean slides and hybridized in oven at 65℃ for 17 hours. After the hybridization, two gene expression wash buffer were prepared. After Milli-Q water wash, we wash the microarray slides with disassembly, 1sth wash and 2nd wash.

**Part 5 Measurements**

Profile AgilentG3_GX_1Color was selected for scan control program. The dye channel was "green", scan region was all scan areas, resolution was 3 um and tiff was 20bit. Then we started the scan using G2565CA Microarray Scanner. After generating the microarray scan images, we extracted tiff images using Agilent Feature Extraction software version 10.7.3. The details of the operations for feature extraction could be found in protocol of Agilent One-Color Microarray-Based Exon Analysis.

**Part 6 Normalization controls**

Raw data were normalized by the Quantile algorithm of the limma package of R 3.2.2. software. The hypothesis was that several lncRNAs and mRNAs could be differentially expressed between salt-sensitive individuals and salt-resistant individuals, which may be related with pathogenesis mechanism of salt sensitivity of blood pressure.
