# Supplementary material for: CeRNA network analysis and functional enrichment of salt sensitivity of blood pressure by weighted-gene co-expression analysis
Source: PeerJ. 2019 Sep 13;7:e7534. doi: 10.7717/peerj.7534 (PMC6746216; doi:10.7717/peerj.7534)
Supplement: Supplemental Information 2 [file peerj-07-7534-s002.docx]

**Supplementary materials**

Supplementary Table 1. Characteristics of participants of microarray.

| Variables | Total | SS | SR | *P-*value |
| --- | --- | --- | --- | --- |
| Number (%) | 20 (100.0) | 10 (50.0) | 10 (50.0) | - |
| Gender, Male (%) | 8 (40.0) | 4 (50.0) | 4 (50.0) | 1.000^#^ |
| Age (years) | 63.85±0.47 | 63.60±2.27 | 64.10±2.18 | 0.563^*^ |
| Hypertension (%) | 10 (50.0) | 5 (50.0) | 5 (50.0) | 1.000^#^ |
| TC (mmol/L) | 5.07(2.73, 6.04) | 5.45(3.53, 6.13) | 3.71(1.48, 5.88) | 0.218^&^ |
| TG (mmol/L) | 1.50(1.18, 2.74) | 1.24(0.97, 1.67) | 2.73(1.27, 4.74) | 0.029^&^ |
| HDL (mmol/L) | 2.51(1.24, 3.68) | 3.20(1.54, 4.03) | 1.63(1.14, 3.10) | 0.089^&^ |
| LDL (mmol/L) | 1.64(1.38, 2.41) | 1.60(1.39, 1.74) | 2.10(1.30, 3.97) | 0.280^&^ |
| GLU (mmol/L) | 5.71(5.29, 6.40) | 5.71(5.35, 6.14) | 5.67(4.74, 6.80) | 0.739^&^ |

Note: statistical testing by #, chi square test; *, paired-samples t test; &,Wilcoxon rank sum test; *P*＜0.05 was considered statistically significant. SS, Salt Sensitive; SR, Salt Resistant.; TC, total cholesterol; TG, triglyceride; HDL, high-density lipoprotein; LDL, low-density lipoprotein; GLU, glucose.

Supplementary Table 2. Top 10 lncRNAs and 17 mRNAs in co-expression network of turquoise module.

| lncRNAs | mRNAs | weight | lncRNAs | mRNAs | weight |
| --- | --- | --- | --- | --- | --- |
| lnc-DNAJC12-3:1 | *PLG* | 0.34 | lnc-LETMD1-1:1 | *LOC388780* | 0.66 |
| lnc-DCAF17-5:1 | *PLG* | 0.31 | lnc-SLC25A26-6:1 | *LOC388780* | 0.66 |
| lnc-DHX57-6:1 | *PLG* | 0.30 | NONHSAT078561 | *LOC388780* | 0.66 |
| ENST00000435049 | *PLG* | 0.29 | lnc-NPY5R-5:1 | *LOC388780* | 0.65 |
| lnc-AC079135.1.1-8:1 | *PLG* | 0.29 | lnc-BORA-14:1 | *LOC388780* | 0.65 |
| lnc-NDFIP2-21:1 | *PLG* | 0.28 | lnc-FBXO10-1:1 | *LOC388780* | 0.65 |
| lnc-CNTN4-3:1 | *PLG* | 0.28 | lnc-SEMA6A-9:1 | *LOC388780* | 0.65 |
| lnc-XRCC6BP1-7:1 | *PLG* | 0.28 | lnc-GJA1-5:1 | *LOC388780* | 0.65 |
| lnc-SLC22A16-3:1 | *PLG* | 0.28 | lnc-DPY19L4-2:1 | *LOC388780* | 0.65 |
| lnc-RNF144B-5:1 | *PLG* | 0.28 | lnc-DTX1-2:1 | *LOC388780* | 0.64 |
| lnc-FBXO10-1:1 | *CCR5* | 0.56 | lnc-LETMD1-1:1 | *FOSL1* | 0.59 |
| lnc-PEPD-1:1 | *CCR5* | 0.55 | lnc-FBXO10-1:1 | *FOSL1* | 0.59 |
| lnc-TMEM202-4:1 | *CCR5* | 0.55 | NONHSAT078561 | *FOSL1* | 0.59 |
| lnc-SLC25A26-6:1 | *CCR5* | 0.55 | lnc-TMEM202-4:1 | *FOSL1* | 0.59 |
| lnc-LETMD1-1:1 | *CCR5* | 0.54 | lnc-PEPD-1:1 | *FOSL1* | 0.59 |
| lnc-RNF144B-5:1 | *CCR5* | 0.54 | lnc-BORA-14:1 | *FOSL1* | 0.58 |
| lnc-RPL7A-2:1 | *CCR5* | 0.54 | lnc-SLC25A26-6:1 | *FOSL1* | 0.58 |
| NONHSAT078561 | *CCR5* | 0.54 | lnc-DPY19L4-2:1 | *FOSL1* | 0.58 |
| lnc-GPR19-2:1 | *CCR5* | 0.54 | lnc-HSP90AA1-12:1 | *FOSL1* | 0.58 |
| lnc-BORA-14:1 | *CCR5* | 0.54 | NONHSAT127886 | *FOSL1* | 0.58 |
| NONHSAT120170 | *TARP* | 0.21 | lnc-GFM1-3:1 | *TKTL1* | 0.22 |
| lnc-NPY5R-5:1 | *ZNF98* | 0.68 | lnc-TMEM202-4:1 | *TKTL1* | 0.22 |
| lnc-SEMA6A-9:1 | *ZNF98* | 0.67 | lnc-ADAMTS7-2:1 | *TKTL1* | 0.22 |
| lnc-GJA1-5:1 | *ZNF98* | 0.67 | lnc-FBXO10-1:1 | *TKTL1* | 0.22 |
| lnc-SLC25A26-6:1 | *ZNF98* | 0.67 | lnc-RPL7A-2:1 | *TKTL1* | 0.22 |
| lnc-IL20RA-2:1 | *ZNF98* | 0.67 | lnc-RP11-439E19.8.1-1:1 | *TKTL1* | 0.22 |
| lnc-LETMD1-1:1 | *ZNF98* | 0.66 | lnc-GPR19-2:1 | *TKTL1* | 0.22 |
| lnc-SMARCAD1-1:1 | *ZNF98* | 0.66 | lnc-PEPD-1:1 | *TKTL1* | 0.22 |
| lnc-DPY19L4-2:1 | *ZNF98* | 0.66 | lnc-AC007401.2.1-5:1 | *TKTL1* | 0.22 |
| lnc-CNOT7-5:1 | *ZNF98* | 0.66 | lnc-TMEM202-4:1 | *SEC14L2* | 0.56 |
| lnc-CNTROB-1:1 | *ZNF98* | 0.66 | lnc-FBXO10-1:1 | *SEC14L2* | 0.56 |
| lnc-FBXO10-1:1 | *GPR68* | 0.47 | lnc-ADAMTS7-2:1 | *SEC14L2* | 0.55 |
| lnc-PEPD-1:1 | *GPR68* | 0.47 | lnc-PEPD-1:1 | *SEC14L2* | 0.55 |
| lnc-TMEM202-4:1 | *GPR68* | 0.47 | lnc-RPL7A-2:1 | *SEC14L2* | 0.55 |
| lnc-RNF144B-5:1 | *GPR68* | 0.47 | lnc-GPR19-2:1 | *SEC14L2* | 0.55 |
| lnc-RPL7A-2:1 | *GPR68* | 0.46 | lnc-RNF144B-5:1 | *SEC14L2* | 0.55 |
| lnc-GPR19-2:1 | *GPR68* | 0.46 | lnc-GFM1-3:1 | *SEC14L2* | 0.54 |
| lnc-ADAMTS7-2:1 | *GPR68* | 0.46 | lnc-BORA-14:1 | *SEC14L2* | 0.54 |
| lnc-SLC25A26-6:1 | *GPR68* | 0.45 | lnc-RP11-1396O13.13.1-3:1 | *SEC14L2* | 0.54 |
| lnc-NDFIP2-21:1 | *GPR68* | 0.45 | lnc-SLC25A26-6:1 | *DNAJB6* | 0.40 |
| lnc-LETMD1-1:1 | *GPR68* | 0.44 | lnc-LETMD1-1:1 | *DNAJB6* | 0.4 |
| lnc-SLC25A26-6:1 | *LINC02210-CRHR1* | 0.35 | lnc-NPY5R-5:1 | *DNAJB6* | 0.4 |
| lnc-LETMD1-1:1 | *LINC02210-CRHR1* | 0.34 | lnc-GJA1-5:1 | *DNAJB6* | 0.4 |
| lnc-GJA1-5:1 | *LINC02210-CRHR1* | 0.34 | lnc-DPY19L4-2:1 | *DNAJB6* | 0.4 |
| lnc-FBXO10-1:1 | *LINC02210-CRHR1* | 0.34 | lnc-DTX1-2:1 | *DNAJB6* | 0.39 |
| lnc-NPY5R-5:1 | *LINC02210-CRHR1* | 0.34 | lnc-IL20RA-2:1 | *DNAJB6* | 0.39 |
| lnc-DTX1-2:1 | *LINC02210-CRHR1* | 0.34 | lnc-ZNF485-5:1 | *DNAJB6* | 0.39 |
| lnc-SEMA6A-9:1 | *LINC02210-CRHR1* | 0.34 | lnc-SEMA6A-9:1 | *DNAJB6* | 0.39 |
| lnc-RPL36A-1:1 | *LINC02210-CRHR1* | 0.34 | lnc-RPL36A-1:1 | *DNAJB6* | 0.39 |
| lnc-CNTROB-1:1 | *LINC02210-CRHR1* | 0.34 | lnc-FBXO10-1:1 | *TRARG1* | 0.49 |
| lnc-DPY19L4-2:1 | *LINC02210-CRHR1* | 0.33 | lnc-SLC25A26-6:1 | *TRARG1* | 0.49 |
| lnc-DTX1-2:1 | *GLIPR1L2* | 0.29 | lnc-TMEM202-4:1 | *TRARG1* | 0.49 |
| lnc-FBXO10-1:1 | *GLIPR1L2* | 0.29 | lnc-PEPD-1:1 | *TRARG1* | 0.49 |
| lnc-GJA1-5:1 | *GLIPR1L2* | 0.29 | lnc-RNF144B-5:1 | *TRARG1* | 0.48 |
| lnc-LETMD1-1:1 | *GLIPR1L2* | 0.28 | lnc-GPR19-2:1 | *TRARG1* | 0.48 |
| lnc-PEPD-1:1 | *GLIPR1L2* | 0.28 | lnc-RPL7A-2:1 | *TRARG1* | 0.48 |
| lnc-SLC25A26-6:1 | *GLIPR1L2* | 0.28 | lnc-LETMD1-1:1 | *TRARG1* | 0.47 |
| lnc-RPL36A-1:1 | *GLIPR1L2* | 0.28 | lnc-ADAMTS7-2:1 | *TRARG1* | 0.47 |
| lnc-TMEM202-4:1 | *GLIPR1L2* | 0.28 | lnc-BORA-14:1 | *TRARG1* | 0.47 |
| lnc-RNF144B-5:1 | *GLIPR1L2* | 0.28 | lnc-FBXO10-1:1 | *PTGER3* | 0.59 |
| lnc-LPHN3-9:1 | *GLIPR1L2* | 0.28 | lnc-SLC25A26-6:1 | *PTGER3* | 0.59 |
| lnc-FBXO10-1:1 | *AOC1* | 0.21 | lnc-LETMD1-1:1 | *PTGER3* | 0.59 |
| lnc-RNF144B-5:1 | *AOC1* | 0.21 | lnc-TMEM202-4:1 | *PTGER3* | 0.59 |
| lnc-TMEM202-4:1 | *AOC1* | 0.21 | lnc-PEPD-1:1 | *PTGER3* | 0.59 |
| lnc-PEPD-1:1 | *AOC1* | 0.21 | NONHSAT078561 | *PTGER3* | 0.59 |
| lnc-ADAMTS7-2:1 | *AOC1* | 0.21 | lnc-BORA-14:1 | *PTGER3* | 0.58 |
| lnc-RPL7A-2:1 | *AOC1* | 0.21 | lnc-DPY19L4-2:1 | *PTGER3* | 0.58 |
| lnc-GPR19-2:1 | *AOC1* | 0.21 | lnc-HSP90AA1-12:1 | *PTGER3* | 0.58 |
| lnc-LETMD1-1:1 | *AOC1* | 0.2 | lnc-RNF144B-5:1 | *PTGER3* | 0.58 |
| lnc-FBXO10-1:1 | *TMEM14B* | 0.36 | lnc-CNTN4-3:1 | *FAM111B* | 0.32 |
| lnc-PEPD-1:1 | *TMEM14B* | 0.36 | lnc-AC007401.2.1-5:1 | *FAM111B* | 0.32 |
| lnc-RNF144B-5:1 | *TMEM14B* | 0.36 | lnc-ADAMTS7-2:1 | *FAM111B* | 0.32 |
| lnc-TMEM202-4:1 | *TMEM14B* | 0.36 | lnc-RPL7A-2:1 | *FAM111B* | 0.32 |
| lnc-RPL7A-2:1 | *TMEM14B* | 0.36 | lnc-TMEM202-4:1 | *FAM111B* | 0.32 |
| lnc-GPR19-2:1 | *TMEM14B* | 0.35 | lnc-NDFIP2-21:1 | *FAM111B* | 0.32 |
| lnc-ADAMTS7-2:1 | *TMEM14B* | 0.35 | lnc-SLC22A16-3:1 | *FAM111B* | 0.32 |
| lnc-NDFIP2-21:1 | *TMEM14B* | 0.35 | lnc-PEPD-1:1 | *FAM111B* | 0.31 |
| lnc-SLC25A26-6:1 | *TMEM14B* | 0.35 | lnc-RNF144B-5:1 | *FAM111B* | 0.31 |
| lnc-CYP7B1-9:1 | *TMEM14B* | 0.35 | lnc-GFM1-3:1 | *FAM111B* | 0.31 |

Supplementary Table 3. Top 10 lncRNAs and 9 mRNAs in co-expression network of blue module.

| lncRNAs | mRNAs | weight | lncRNAs | mRNAs | weight |
| --- | --- | --- | --- | --- | --- |
| lnc-GHDC-1:1 | *CLEC1A* | 0.22 | ENST00000469070 | *CLEC1A* | 0.19 |
| ENST00000522547 | *CLEC1A* | 0.22 | NR_030349 | *CLEC1A* | 0.18 |
| lnc-LYN-4:1 | *CLEC1A* | 0.22 | ENST00000608069 | *CLEC1A* | 0.15 |
| ENST00000500185 | *CLEC1A* | 0.20 | ENST00000617424 | *CLEC1A* | 0.14 |
| lnc-DNAJC25-GNG10-1:1 | *CLEC1A* | 0.20 | lnc-CR2-2:1 | *CLEC1A* | 0.14 |
| lnc-KCNRG-1:1 | *CLEC1A* | 0.18 | lnc-PTEN-7:1 | *CLEC1A* | 0.13 |
| lnc-SCLY-1:7 | *CLEC1A* | 0.18 | lnc-IL1A-3:1 | *CLEC1A* | 0.13 |
| lnc-RP11-712L6.5.1-2:4 | *CLEC1A* | 0.18 | ENST00000435967 | *CLEC1A* | 0.13 |
| NR_039636 | *CLEC1A* | 0.18 | NM_033272 | *CLEC1A* | 0.13 |
| ENST00000605056 | *CLEC1A* | 0.17 | lnc-LRIG2-1:1 | *CLEC1A* | 0.12 |
| lnc-GHDC-1:1 | *IL1B* | 0.32 | lnc-RP11-712L6.5.1-2:4 | *DHRS13* | 0.31 |
| lnc-DNAJC25-GNG10-1:1 | *IL1B* | 0.30 | lnc-SEC22C-4:1 | *DHRS13* | 0.31 |
| lnc-IL1A-3:1 | *IL1B* | 0.29 | lnc-RP11-1220K2.2.1-1:2 | *DHRS13* | 0.30 |
| lnc-CR2-2:1 | *IL1B* | 0.28 | lnc-DNAJC25-GNG10-1:1 | *DHRS13* | 0.30 |
| lnc-RP11-712L6.5.1-2:4 | *IL1B* | 0.27 | lnc-GHDC-1:1 | *DHRS13* | 0.29 |
| lnc-SEC22C-4:1 | *IL1B* | 0.27 | lnc-FLOT2-2:1 | *DHRS13* | 0.29 |
| ENST00000605056 | *IL1B* | 0.27 | ENST00000439443 | *DHRS13* | 0.28 |
| NM_033272 | *IL1B* | 0.27 | NM_001293626 | *DHRS13* | 0.28 |
| ENST00000419160 | *IL1B* | 0.26 | lnc-SOX6-3:2 | *DHRS13* | 0.27 |
| NR_033839 | *IL1B* | 0.26 | NR_040079 | *DHRS13* | 0.27 |
| lnc-RP11-712L6.5.1-2:4 | *ITGB4* | 0.28 | lnc-GHDC-1:1 | *KCNH7* | 0.33 |
| ENST00000439443 | *ITGB4* | 0.27 | lnc-RP11-712L6.5.1-2:4 | *KCNH7* | 0.33 |
| ENST00000419160 | *ITGB4* | 0.26 | lnc-DNAJC25-GNG10-1:1 | *KCNH7* | 0.32 |
| lnc-DNAJC25-GNG10-1:1 | *ITGB4* | 0.25 | ENST00000605056 | *KCNH7* | 0.31 |
| lnc-GHDC-1:1 | *ITGB4* | 0.24 | ENST00000439443 | *KCNH7* | 0.29 |
| ENST00000605056 | *ITGB4* | 0.21 | lnc-KCNRG-1:1 | *KCNH7* | 0.27 |
| lnc-KCNRG-1:1 | *ITGB4* | 0.21 | ENST00000419160 | *KCNH7* | 0.27 |
| NR_033839 | *ITGB4* | 0.20 | lnc-CR2-2:1 | *KCNH7* | 0.27 |
| NR_040079 | *ITGB4* | 0.19 | lnc-PAG1-2:1 | *KCNH7* | 0.26 |
| lnc-CR2-2:1 | *ITGB4* | 0.19 | lnc-RP11-1220K2.2.1-1:2 | *KCNH7* | 0.26 |
| lnc-GHDC-1:1 | *LOC643802* | 0.36 | lnc-LYN-4:1 | *KIF6* | 0.23 |
| lnc-KCNRG-1:1 | *LOC643802* | 0.32 | NR_030349 | *KIF6* | 0.22 |
| lnc-DNAJC25-GNG10-1:1 | *LOC643802* | 0.30 | ENST00000608069 | *KIF6* | 0.20 |
| ENST00000605056 | *LOC643802* | 0.27 | ENST00000510449 | *KIF6* | 0.20 |
| lnc-RP11-712L6.5.1-2:4 | *LOC643802* | 0.26 | NM_001297750 | *KIF6* | 0.20 |
| ENST00000522547 | *LOC643802* | 0.26 | ENST00000469070 | *KIF6* | 0.20 |
| lnc-CR2-2:1 | *LOC643802* | 0.25 | NR_039636 | *KIF6* | 0.19 |
| lnc-COPZ1-2:1 | *LOC643802* | 0.25 | ENST00000500185 | *KIF6* | 0.18 |
| lnc-PAG1-2:1 | *LOC643802* | 0.24 | ENST00000522547 | *KIF6* | 0.17 |
| ENST00000439443 | *LOC643802* | 0.22 | ENST00000435967 | *KIF6* | 0.16 |
| lnc-RP11-712L6.5.1-2:4 | *MGAM2* | 0.31 | ENST00000510449 | *SCN9A* | 0.20 |
| ENST00000419160 | *MGAM2* | 0.28 | lnc-LYN-4:1 | *SCN9A* | 0.20 |
| lnc-RP11-1220K2.2.1-1:2 | *MGAM2* | 0.27 | NR_030349 | *SCN9A* | 0.20 |
| ENST00000439443 | *MGAM2* | 0.26 | lnc-RP11-712L6.5.1-2:4 | *SCN9A* | 0.18 |
| lnc-SEC22C-4:1 | *MGAM2* | 0.26 | NM_144683 | *SCN9A* | 0.17 |
| NR_040079 | *MGAM2* | 0.25 | NR_039636 | *SCN9A* | 0.17 |
| ENST00000605056 | *MGAM2* | 0.25 | NM_001297750 | *SCN9A* | 0.16 |
| ENST00000617424 | *MGAM2* | 0.25 | lnc-AL035696.1-7:1 | *SCN9A* | 0.16 |
| lnc-DNAJC25-GNG10-1:1 | *MGAM2* | 0.25 | ENST00000500185 | *SCN9A* | 0.16 |
| NR_033839 | *MGAM2* | 0.23 | ENST00000522547 | *SCN9A* | 0.16 |


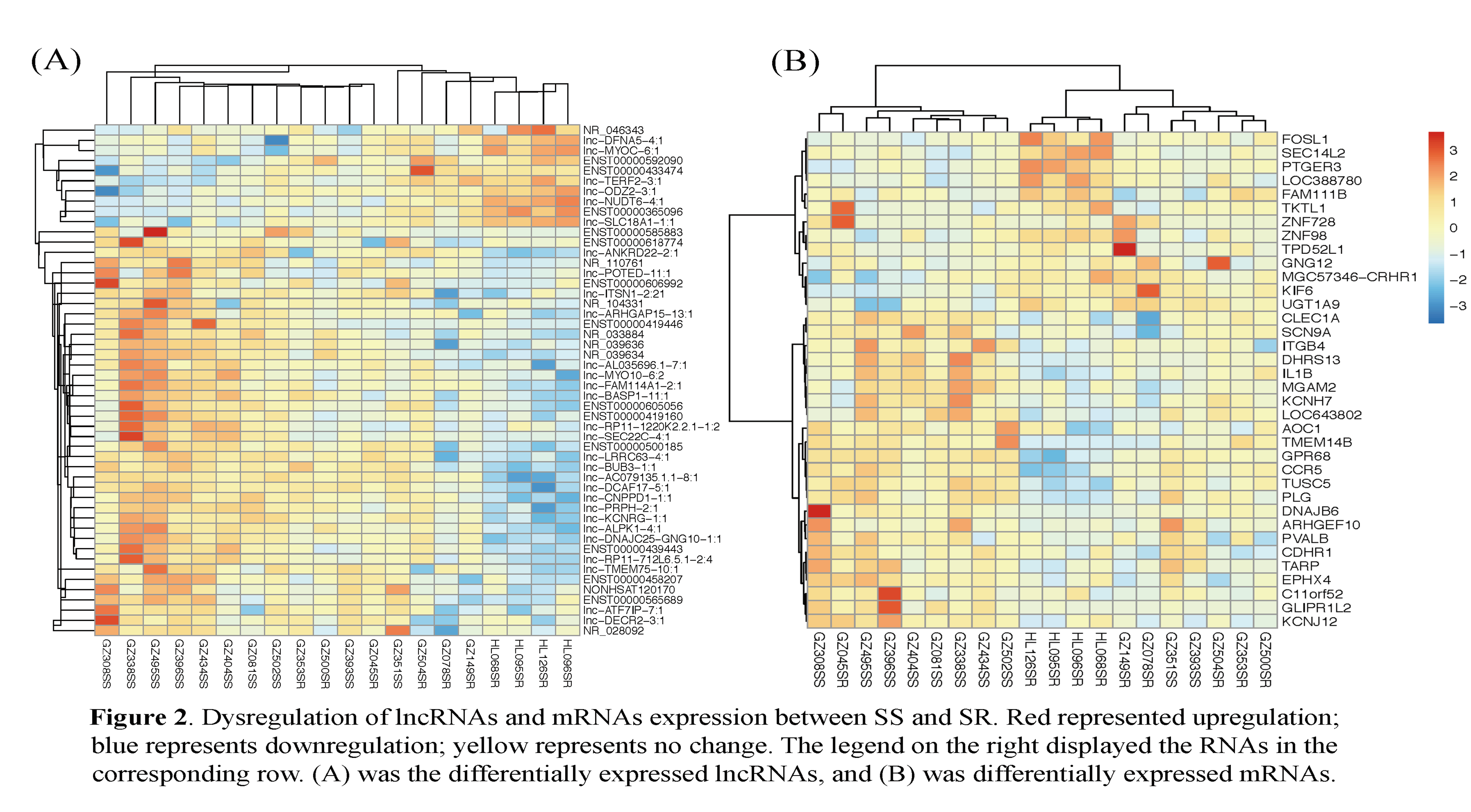


Supplementary Figure 1. Dysregulation of lncRNAs and mRNAs expression between SS and SR. Red represented upregulation; blue represents downregulation; yellow represents no change. The legend on the right displayed the RNAs in the corresponding row. (A) was the differentially expressed lncRNAs, and (B) was differentially expressed mRNAs (top 50).


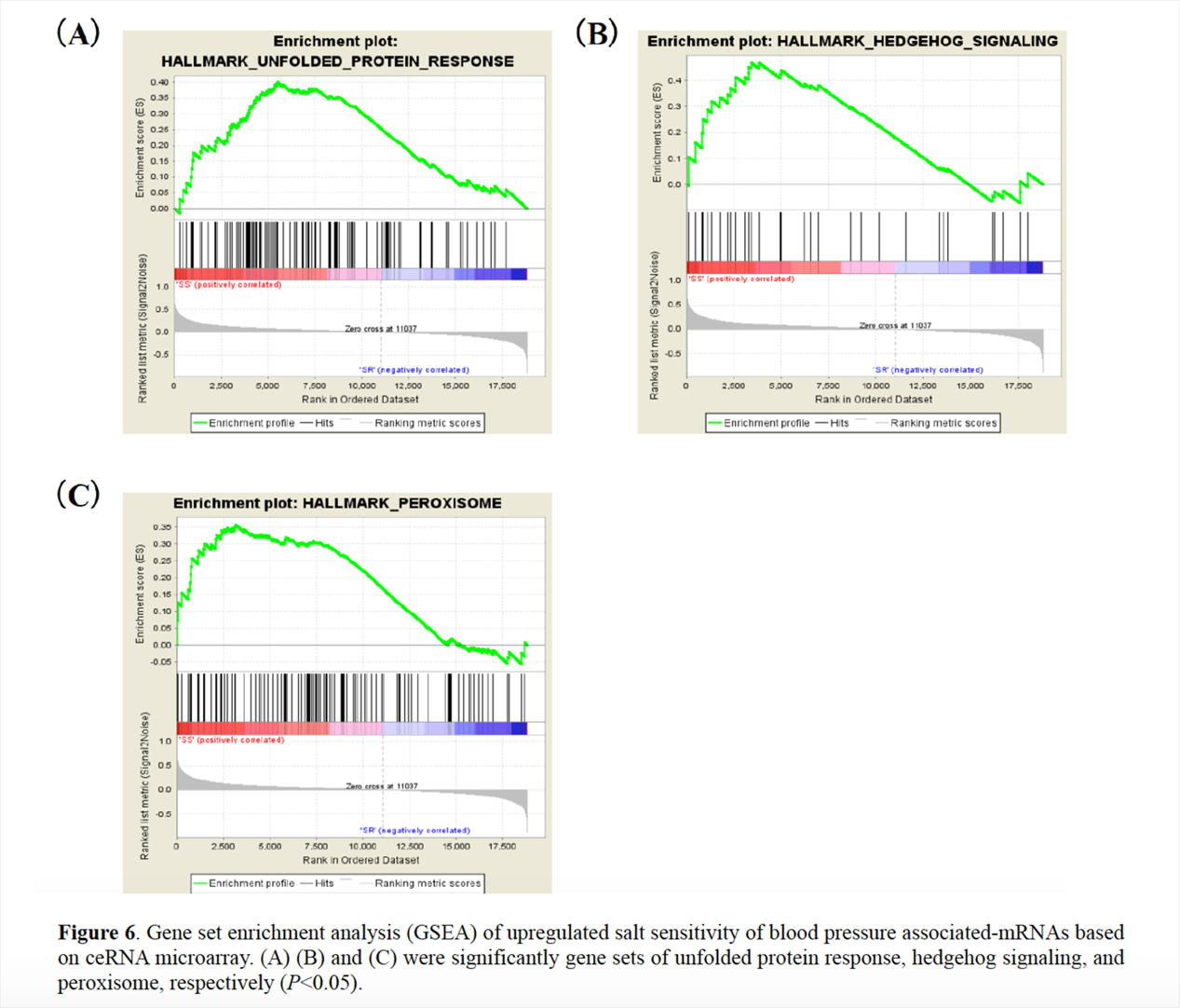


Supplementary Figure 2. Gene set enrichment analysis (GSEA) of upregulated salt sensitivity of blood pressure associated-mRNAs based on ceRNA microarray. (A) (B) and (C) were significantly gene sets of unfolded protein response, hedgehog signaling, and peroxisome, respectively (*P*<0.05).
